# Evaluation of Ozone Application in Dental Unit Water Lines Contaminated with Pathogenic Acanthamoeba

**Published:** 2015

**Authors:** Wafaa HIKAL, Basma ZAKI, Hany SABRY

**Affiliations:** 1 *Parasitology Lab, Water Pollution Research Department, National Research Centre, Egypt*; 2 *Oral Medicine and Periodontology, Surgery and Oral Medicine Department, National Research Centre, Egypt*; 3 *Laser applications in Fixed Prosthodontics, Prosthodontics Research Department, National Research Centre, Egypt*

**Keywords:** *Acanthamoeba* spp., Dental units water lines, PCR, Ozone

## Abstract

***Background:*** In this study morphological and molecular characterization of *Acanthamoeba* strains, isolated from dental unit waterlines (DUWLs) were surveyed and the levels of disinfection achievable in vitro by the application of ozone disinfectant to DUWLs were evaluate.

***Methods:*** Water samples were collected from air-water syringes, cup fillers and tap water before and at the end of the working day. They were cultured on non-nutrient agar (NNA) plates. Species identification was carried out with a PCR assay based on sequence analysis of the 18S rRNA gene. The cellular response to ozone was tested on *Acanthamoeba* cyst with different doses at different contact time in vitro twice.

***Results:*** Prevalence rates for *Acanthamoeba* contamination were 100, 100 and 72% for air-water syringes, cup fillers and tap water, respectively. The morphological analysis revealed the presence of* A. castellanii, A. griffin, A. hatchitti* and* A. lenticulata*. Phylogenetic analysis of the sequences showed the four strains to be closely related to a sequence type (T3, T4, T5 and T11).* Acanthamoeba* cells were stained with trypan blue, which revealed killed of *Acanthamoeba* instantaneously after 10 minutes in ozonized water. There was no growth of *Acanthamoeba* occurred after ozone treatment in water bottles for 5 minutes with a flow rate of 500 mg/hour.

***Conclusion*** *:* Ozone can play an important role in controlling the problem of contamination of DUWLs as a potent disinfectant.

## Introduction

Free-living amoebae (FLA) are opportunistic and ubiquitous protozoa that have a cosmopolitan distribution in the environment"(1). Among the many genera of this FLA,* Acanthamoeba *species were recognized to cause human diseases, which are responsible for opportunistic and non-opportunistic infections in humans and other animals (2, 3).* Acanthamoeba *species have been isolated from many different sources, such as freshwater, seawater, chlorinated water from swimming pools, dental units, and contact lens cases. The genus *Acanthamoeba* is the causative agent of granulomatous amoebic encephalitis (GAE). Amoebic encephalitis is a life-threatening disease of the central nervous system (CNS), which occurs in immune-compromised patients, while keratitis is reported in healthy individuals (4).

In addition to disease caused by direct exposure to *Acanthamoeba* spp., recently, it is proposed that *Acanthamoeba* might play a role in the increased incidence of nosocomial infections (5). *Acanthamoeba* act as natural vectors or reservoirs for phylogentically diverse microorganisms while some of them replicate intra-cellularly, such as *Escherichia coli, Klebsiella, Bacillus *spp*., Mycoplasma, Legionella pneumophila, Mycobacterium avium, M. leprae, Clostridium frigidicarnis, Porphyromonas gingivalis, Prevotella intermedia, Burkholderia pseudomallei, Afipiafelis, Vibrio cholerae, Mobiluncus curtissi, Campylobacter* spp*., Helicobacter pylori*, *Cryptococcus neoformans, Candida *spp*., Coxiellaburnetti, Chlamydia, Rickettsia, *Coxsackievirus, Adenovirus and Norovirus (5-10). Many pathogenic microorganisms that replicate inside free-living amoebae, such as viruses and/or bacteria are more virulent and more resistant, complicating the situation when FLA and other pathogens co-exist in health care settings(11).While amoeba normally feed on bacteria, Rowbotham (12) reported that these bacteria survive inside amoeba, a finding that has been linked to the fact that the natural human targets of *Legionellae* infection macrophages and other phagocytic cells are amoeboid cells. In such cases, amoebae escape infection by the bacteria through encystment (12). Consequently, adaptation to amoeba (i.e., “amoeba-resistance”) served evolutionarily as a pre-adaptation to the macrophage internal environment, an important step in the process of becoming a human pathogen (10, 13, 14). There are at least four ways in which waterborne microorganisms might cause infection in a patient undergoing dental work: hematogenous spread during surgical procedures, local mucosal (oral or conjunctival) contact, ingestion and inhalation (15).

Barbeu (16) presented a case report of a woman with contact lenses who visited her dentist for replacement of a bridge. The case report highlighted the risk that may be associated with *Acanthamoeba* in the water of a dental unit. During the treatment, a stream of water was directed from the hand piece into her right eye. A microbiological examination nearly 2 months later revealed amoebae in corneal samples. Because of subsequent pain in the eye, the patient consulted several ophthalmologists, who discovered abrasive lesions of the cornea and inflammation.

The quality of DUWLs is of considerable importance since patients and dental staff are regularly exposed to water and aerosols generated from the dental unit. The unique feature of dental chair water lines is the capacity for rapid development of a biofilm on the dental water supply lines combined with the generation of potentially contaminated aerosols. Dental water may be ingested, inhaled in the form of aerosols or directly contaminate surgical wounds. Dentists have a duty of care to their staff and patients. It is deemed ethically unacceptable to expose knowingly patients to contaminated water (17). There is no evidence of a widespread public health problem from exposure to DUWLs. Nevertheless, the goal of infection control is to minimize the risk from exposure to potential pathogens and to create a safe working environment in which to treat patients. The ever increasing number of patients who are either immune-compromised or immune-suppressed due to drug therapy, alcohol abuse or systemic disease has produced a cohort of patients susceptible to environmental waterborne opportunistic pathogens such as fungi, free living amoebae, protozoa and nematodes as well as the consistently reported recovery of saprophytic and opportunistic gram negative pathogens (18). Ozone is a tri-atomic form of oxygen and is characterized by a high oxidation potential that conveys bactericidal and viricidal properties (19-21). It is a powerful broad-spectrum antimicrobial agent active against bacteria, fungi, viruses, protozoa and fungal spores (22). It can break cell membrane or protoplasm, inhabilitating cellular reactivation of bacteria, coliforms, viruses and protozoa (23). Ozone is widely used as a disinfectant in drinking water and wastewater treatment. Ozone is 1.5-times stronger than chlorine and is effective over a much wider spectrum of microorganisms than chlorine and other disinfectants (24). Protozoan organisms disrupted by ozone include *Giardia*, *Cryptosporidium*, and free-living amoebas, namely *Acanthamoeba*, *Hartmonella,* and *Naegleria*, the anit-protozoal action has yet to be elucidated. In Egypt, only a few studies on *Acanthamoeba* have been published. 

The aim of this study was therefore first; detect the presence of *Acanthamoeba* species in dental water unit morphologically and by polymerase chain reaction. Second, identification of *Acanthamoeba* isolates at the species level by sequence analysis. Third, evaluate the levels of disinfection achievable in vitro and by the application of ozone disinfectant to DUWLs.

## Materials and Methods

***Sample collection***

Fifty water samples collected during the period from the beginning of January, 2013 to the end of December, 2013 from DUWLs at the medical services unit of National Research Center, Dokki, Giza, Egypt. Two different collects were realized from dental hand pieces, air-water syringes and cup fillers. The first collect in the early morning before materials flush and patient consultations. The second collect was taken at the end of the working day. At the same time, samples from tap water (used for hand washing and connected to the same water supply as the DUWLs) were realized using sterile containers. After collection, water samples were taken immediately to the parasitology laboratory, Water Pollution Research Department, National Research Center for analysis and culture.

Overall, 500 mL of each water sample was filtered through cellulose acetate filter 0.45 μm porosity under a weak vacuum. Filters were inoculated on to NNA plates overlaid with *E. coli*. Plates were incubated at 37and 40 ^°^C and observed daily for the presence of amoebae as previously described (25). The genus *Acanthamoeba *was identified from other free-living amoebae based on its distinctive feature of trophozoites and cysts, particularly the double walled cyst shape. For the classification, the Pussard & Pons (26) and Page (27) keys were applied. Axenic cultures were obtained by transferring a piece of agar containing some amoeba to liquid culture medium peptone yeast-extract glucose (PYG) slightly modified (28).

***Extraction of nuclear DNA***

The cells collected from liquid culture PYG were centrifuged (500 xg) for 10 min at 4 °C and washed for three times with PBS (phosphate-buffered-saline) pH 7.2, then lysed by treatment with lysozyme and freeze-thawing (using liquid nitrogen and heating to 65 °C). The samples were then treated with proteinase K, SDS and RNAse. The DNA was purified using the QIAamp DNA mini kit (Qiagen) according to the manufacturer's instructions** (**29).

***PCR amplification***

To confirm the identity of *Acanthamoeba*, PCR reactions were performed using genus specific primers as previously described (30). DNA was used as the template for PCR. Primer sequences were 5-GGCCCAGATCGTTT-ACCGTGAA-3 and 5^-^TCTCACAA-GCTGCTA-GGGAGTAC-3. PCR was performed in a volume of 50 µl containing1.25 U Taq polymerase (Qiagen), 0.1–1.0 ng DNA, 200 µM dNTPs,4 mM MgCl_2_ and 0.5 µM primer. PCR reactions were performed at 94 ^°^C for 1 min, 55^°^C for 1 min and 72 °C for 2 min for 35 cycles, with a final elongation step of 10 min at 72 ^°^C. Amplified DNA was electrophoresed on a 1.5% agarose gel.

***Sequence and phylogenetic analysis***

The PCR products were purified using QIA-quick Gel Extraction Kit. (Qiagen, Cat. No. 28704).Sequencing of the PCR product was performed with a Prism Big Dye v3.1 kit (Applied Biosystems, Cat. No. 4336917) on an ABI 310 DNA automated sequencer (Applied Biosystems).The obtained sequences were aligned using Mega 3.0 software program (31).

***Determination of the accurate ozone concentration***

Ozone concentration was determined using the semi-batch method. Different ozone concentrations in water were attained by adjusting the flow rate of gaseous ozone in the double distilled water for a specific time according to Kim and Yousef (32). Ozone generator for disinfection type N 1888A, China was used in the disinfection procedures with an ozone rate of 500 mg/hour. The ozonizer was mounted in a position higher than the water level to avoid the water back flow into the ozone generator. Different ozone concentrations were used to reach the effective concentration capable of killing *Acanthamoeba* (Table 1).

***Ozone treatment in vitro***

Amoebae from several agar plates were harvested by washing each agar surface with 3 ml of sterile pages amoeba saline followed by centrifugation at 500 xg for 10 min. After centrifugation, supernatants were discarded and the pellet was suspended in sterile 0.01 M phosphate buffer (pH 7.0). They were then washed three times in the same buffer, counted with a hemocytometer, and re-suspended to a final concentration of 10^3^cells/ml (33, 34). *Acanthamoeba* cysts were treated with various concentrations of ozone as shown in (table 2).

**Table 1:** Different ozone concentrations and time used for killing *Acanthamoeba*

**Time (min)**	**Ozone concentration (mg)**
1	8.5
2	17
3	25.5
4	34
5	42.5
6	51

Ozone density (g/L): 1.429 at 0^ o^C and 760 mmHg

A total of 100 µl of the washed amoebae was added to each flask, containing 50 ml of the required concentration of ozonized water and incubated for 10 min. After exposure time, the amoeba suspension was centrifuged at 500 xg for 10 minutes, the supernatant discarded and the pellet divided into two parts. One part of samples was stained with trypan blue and counted microscopically using hemocytometer to determine the number of dead amoeba after exposure to ozone. Trypan blue is a vital stain used to color selectively dead tissues or cells blue. Live cells or tissues with intact cell membranes are not colored. Hence, dead cells are shown as a distinctive blue color under a microscope. Since live cells are excluded from staining.

 A second part of samples was inoculated onto agar plates overlaid with *E. coli* and incubated at 30°C for 7 days to determine the viability of amoeba after exposure to ozone (33, 34). Agar plate cultures were examined by inverted microscope as mentioned above (25). 

***Application of ozone in dental unit water lines (DUWLs)***

Two DUWLs were treated with O_3 _and subjected to *Acanthamoeba* assessment. It was connected to the dental unit water bottles for 5 minutes in the first unit and for 10 minutes in the second unit. The lines were subsequently flushed with ozonized water for 5 minutes, after which water was immediately sampled via the high-speed hand piece into sterile containers. This was repeated daily every morning for 7 days. Water samples were collected before and after treatment. All the samples were cultured on nutrient agar plates and incubated at 37 °C for 7 days to determine the presence of *Acanthamoeba* before and after exposure to ozone (35).

## Results

***Prevalence of Acanthamoeba in DUWLs***

*Acanthamoeba* species were isolated from 100 of 100 water samples collected from hand pieces, cup fillers and air-water syringes, with an overall point prevalence of 100% (Table 2), but they were detected only from 36 0f 50 (72%) water samples collected from tap water at the same time.

**Table 2:** Prevalence of *Acanthamoeba* species in water samples from DUWLs at the two sampling time

**Percentage of samples positive for ** ***Acanthamoeba***						
**Sampling Sites**	**1** ^st^ ** Collect**		**2** ^nd^ ** Collect**		**Total**	
	**n**	**%**	**n**	**%**	**n**	**%**
Tap water	20/25	80	16/25	64	36/50	72
Cup Filler	25/25	100	25/25	100	50/50	100
Air-Water Syringe	25/25	100	25/25	100	50/50	100

**Fig. 1:**
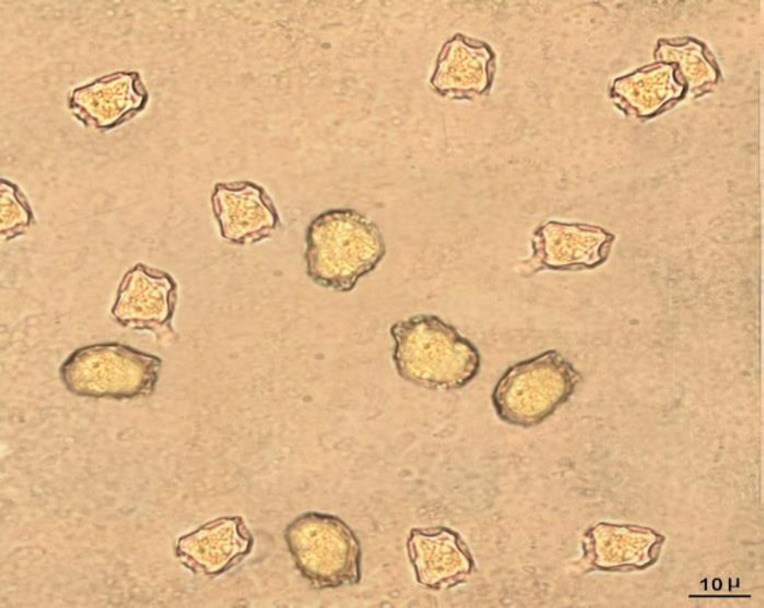
*Acanthamoeba*species in agar plates

*Acanthamoeba* were isolated based on morphological characteristics of trophozoites and cysts. Overall, these results suggested a wide distribution of* Acanthamoeba* in DUWLs (Fig. 1).

 The identity of *Acanthamoeba* isolates was further confirmed using PCR analysis as described above. *Acanthamoeba *were successfully cultured in PYG medium containing penicillin (100 U/ml) and streptomycin (100 g/ml) at 30 ^°^C.

***Molecular Study***

**Fig. 2:**
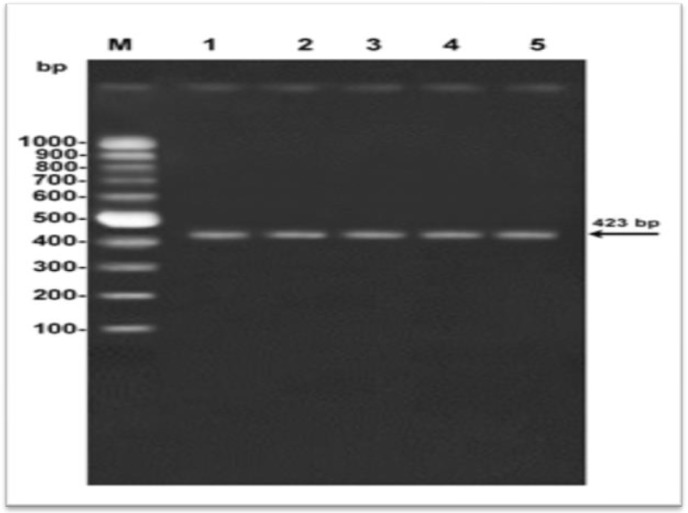
Agarose gel electrophoresis showing amplification of 18S rDNA of different *Acanthamoeba *isolates were subjected to electrophoresis on 1.5% agarose gel parallel containing ethidium bromide to 100 bp DNA ladder. M: DNA ladder;1-5:*A. *isolates

PCR amplification, with genus specific primers of the all samples identified as *Acanthamoeba *spp., showed the presence of a band in between of 423-551 bp (Fig. 2).

***Sequence analysis***

**Fig. 3:**
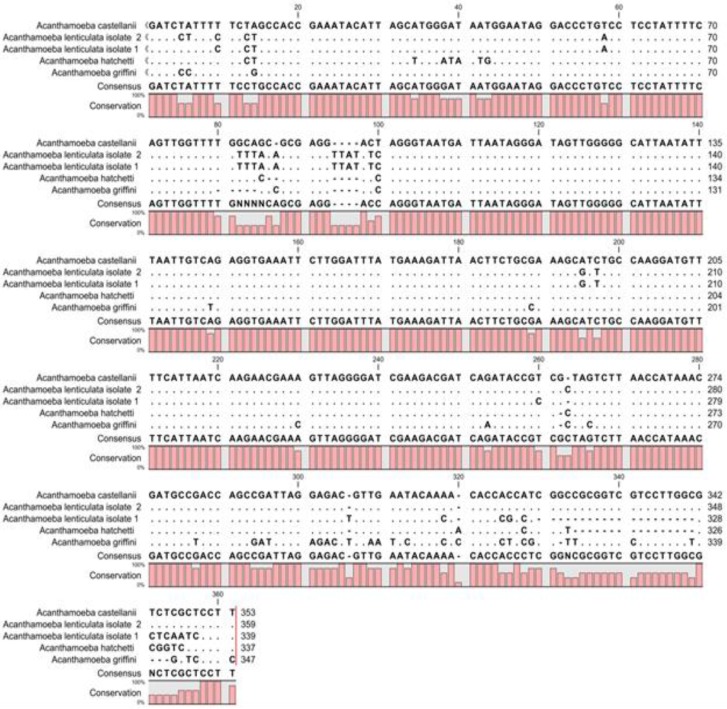
Sequence alignment showing genetic variation among *Acanthamoeba* isolates.

A portion of the 18S rRNA gene (between 423 and 551 bp in length) was amplified and sequenced for five of the *Acanthamoeba *strains isolated. Sequence analysis using a BLAST search indicated an identity of >98% with *Acanthamoeba *18SrRNA gene reference sequences (calculation based on the p-distance, Mega version 2b3). *Acanthamoeba* isolates investigated in this study were included in sequence types (T3, T4, T5 and T11) (Fig. 3).

Dashes (-) indicate gaps, and dots (.) indicate bases that are identical to that of the genotype sequence

***Effect of ozone on Acanthamoeba in vitro***

**Fig.4:**
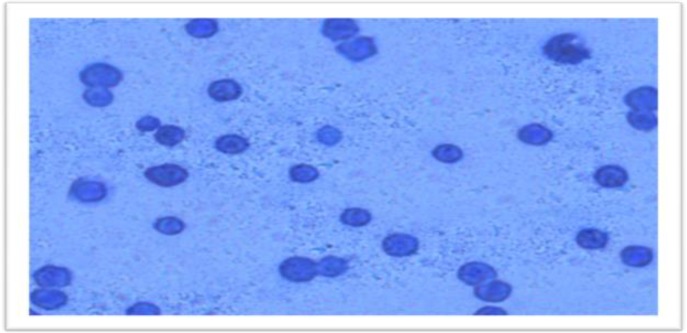
*Acantamoeba*species stained with trypan blue after 5 minutes of exposure to ozonized water.

To examine the effect of ozone as a disinfectant product, *Acanthamoeba *isolates were exposed to several concentrations of ozonized water. The cell viability of *Acanthamoeba* cyst decreased to 52% after exposure to ozonized water for 4 minutes while *Acanthamoeba* was killed (100%) very rapidly after 5 minutes of exposure and rupture occurred in the cell wall as shown in Fig.4. Agar plate gave comparable results, where no growth was occurred.

***Application of ozone in DUWLs bottles***

Before any treatment, agar plate was positive for *Acanthamoeba* growth in examined units, while after treatment with ozone in the 1^st^ unit after 5 minutes after exposure to ozone growth took place after 3 days of incubation. In the 2^nd ^unit, after exposure to 10 minutes of ozone, there is no growth occur during the 7 days of culture.

## Discussion

To our knowledge, this is the first study reporting on the morphological, molecular detection and identification of genus* Acanthamoeba *at the species level in Egypt. In addition, the use of ozone as a disinfectant in vitro and its application in the dental unit water lines was performed for the first time in Egypt.

Free-living amoebae, such as the genus *Acanthamoeba,* have been commonly found in various environments all around the world and recognized as important pathogens of humans or animals (28).* Acanthamoeba *species are opportunistic pathogens of immune-suppressed people and mostly cause GAE. The pathology of disease can be observed in the lungs, sinuses and skin in immune-deficient patients (36, 37). Additionally, *Acanthamoeba *species (spp.) invade the cornea of the eye and cause *Acanthamoeba* keratitis (AK) due to contact lens usage (12, 38-42). Besides their pathogenicity, *Acanthamoeba* may transfer some other pathogens to the human body (43).

Bacterial biofilms may be the source of increased populations of amoebic protozoa in the tubing of dental units (12). They play an important role in the pathogenesis of *Acanthamoeba*, particularly, in keratitis infections. They provide the parasite an attractive niche and an abundant nutrient. The concentration of organisms in these biofilms is almost 300 times higher than what is found in tap water (12). Chappel et al. (44) showed that more than 80% of normal human populations have *Acanthamoeba* antibodies. Therefore, *Acanthamoeba* pathogenicity was the resultant of several processes, which must occur together and depends on its capacity of mucous adhesion and tissue migration. This is due to their capacity to resist chemical and physical treatments used for drinking water production and distribution (45, 46) they can colonize virtually any artificial water system. GAE is relatively rare. To date, approximately 150 cases have been described worldwide. Because of the problems in recognizing GAE, it is possible that other cases occurred but were undiagnosed or misdiagnosed. 

One of the interesting findings in our study was the isolation of group number (II), sequence type (T4, T3 and T11) for *A. castellanii, A. griffin* and *A. hatchitti, *respectively. Also group number (III), sequence type (T5) for *A. lenticulata* (18) genotypes of genus *Acanthamoeba* reviewed in (6, 47) from DUWLs for the 1^st^ time in Egypt.T4 genotype has been most frequently associated with blinding keratitis and fatal GAE, which occurs mostly in immune-compromised individuals. *Acanthamoeba* *keratitis* is a painful sight-threatening ulceration of the cornea and is most likely associated with the improper use of contact lenses (6). Despite the occasional cases of *Acanthamoeba* *keratitis* due toT3 (48), and T11 (49), the majority of cases are due to the T4 genotype. In France, a single and fatal case of *Acanthamoeba lenticulata* GAE, with extra-cerebral dissemination, in a heart transplant patient was reported (50). T4 was widely distributed in the environmental samples isolated from the DUWLs. These results, together with previous findings, suggest that *Acanthamoeba *isolates belonging to the T4 genotype naturally occur in the environment and present potential reservoirs and therefore sources of infection to susceptible hosts.

In Egypt, Hassan et al., (51) reported *Acanthamoeba *species contaminating hemodialysis and dental units in Alexandria.* Acanthamoeba *spp. have also been reported in dental unit water lines (12, 1). Importantly, the endocytobionts pathogenic bacteria within amoebae could be a source of microbiological risk for patients with deep dental work or immune-suppression (1).

There is no evidence of a widespread public health problem from exposure to DUWLs. Nevertheless, the goal of infection control is to minimize the risk from exposure to potential pathogens and to create a safe working environment for both the patients and the dental staff members (52).

Ozone at a concentration of 0.5 mg/L also reduced planktonic amoebae in a pipe system with an established biofilm, but it was unable to eradicate the organisms from the system and allowed them to rebound quickly once treatment was stopped (53). In a separate study, ozone concentrations up to 1.7 mg/L reduced the amoebae population by 1 to 2 logs (45). Ozone at an initial concentration of 6.75 mg/L has been effective at reducing but not completely removing *Acanthamoeba* trophozoites (34).

We recommended that patients wear safety glasses during dental treatments. The hand piece was inadvertently activated before being placed in the patient’s mouth. Staff in dental clinics should drain the waterlines of each dental unit every morning for several minutes and for 30 to 45 seconds between patients. Doing so reduces the concentrations of bacteria and amoebae in the water by 96% and 66%, respectively. Every morning, remove the hand pieces, the air and water syringes, and the end fitting on the ultrasound scalar and then flush each waterline with fresh water. After each patient, the hand pieces should be turn on at high speed for 20 to 30 seconds to evacuate all air and water. Use sterile water or a sterile saline solution to rinse surgical wounds or to cut bones during surgery. When using bottled water or another water supply system, follow the manufacturer’s instructions for daily and weekly maintenance.

## Conclusion

The presence of potentially pathogenic *Acanthamoeba *in dental unit waterlines is of a great concern. Molecular techniques such as PCR and sequence analysis were effective and successful for the detection of different* Acanthamoeba* spp. The use of ozone in dental units is effective and recommended to be routinely used.

## References

[1] Trabelsi H, Sellami A, Dendena F, Sellami H, Chelkh-Rouhou F, Makni F, Ben DS, Ayadi A (2010). Free-living amoebae (FLA): Morphological and molecular identification of Acanthamoeba in dental unit water. Parasite.

[2] Siddiqui R, Khan NA (2012). Biology and pathogenesis of Acanthamoeba. Parasites &Vectors.

[3] Lorenzo-Morals J, Martín-Navarro C, López-Arencibia A, Arnalich-Montiel F, Piñero JE, Valladares B (2013). Acanthamoeba keratitis: an emerging disease gathering importance worldwide?. Trends Parasitol.

[4] Marciano-Cabral F, Puffenbarger R, Cabral GA (2000). The increasing importance of Acanthamoeba infections. J Eukaryot Microbiol.

[5] Marciano-Cabral F, Cabral G (2003). Acanthamoeba spp. as agents of disease in humans. Clin Microbiol Rev.

[6] Waldner H, Collins M, Kuchroo VK (2004). Activation of antigen-presenting cells by microbial products breaks self-tolerance and induces autoimmune disease. J Clin Invest.

[7] Khan NA (2006). Acanthamoeba: biology and increasing importance in human health. FEMS Microbiol Rev.

[8] Mattana A, Serra C, Mariotti E, Delogu G, Fiori PL, Cappuccinelli P (2006). Acanthamoeba castellanii promotion of in vitro survival and transmission of coxsackie b3 viruses. Eukaryot Cell.

[9] Thomas V, McDonnell G, Denyer SP, Maillard JY (2009). Free-living amoebae and their intracellular pathogenic microorganisms: risks for water quality. FEMS Microbiol Rev. Technol.

[10] Scheid P (2014). Relevance of free-living amoebae as hosts for phylogenetically diverse microorganisms. Parasitol Res.

[11] Barbeau J, Buhler T (2001). Biofilms augment the number of free-living amoebae in dental unit waterlines. Res Microbiol.

[12] Rowbotham TJ (1986). Current views on the relationships between amoebae, Legionellae and man. Isr J Med Sci.

[13] Greub G, Raoult D (2004). Microorganisms resistant to free-living amoebae. Clin Microbiol Rev.

[14] Yan L, Cerny RL, Cirillo J (2004). Evidence that hsp90 is involved in the altered interactions of Acanthamoeba castellanii variants with bacteria. Eukaryot Cell.

[15] Barbeau J (2000). Waterborne Biofilms and Dentistry: The Changing Face of Infection Control. J Can Dent Assoc.

[16] Barbeau J Lawsuit against a Dentist Related to Serious Ocular Infection Possibly Linked to Water from a Dental Hand piece JCDA.

[17] US Department of Health and Human Services (1993). Recommended infection control practices for dentistry. MMWR.

[18] Williams JF, Johnston AM, Johnson B (1993). Microbial contamination of dental unit waterlines: prevalence, intensity and microbial characteristics. JADA.

[19] Burleson GR, Murray TM, Pollard M (1975). Inactivation of viruses and bacteria by ozone with and without sonication. Appl Microbiol.

[20] Horvath M, Bilitzky L, Huttner J. Ozone (1985). Elsevier, New York, 350 p. Khadre, MA, Yousef A.E, 2001. Sporicidal action of ozone and hydrogen peroxide: a comparative study. Int J Food Microbiol.

[21] Kim JG, Yousef AE, Dave S (1999). Application of ozone for enhancing the microbiological safety and quality of foods: a review. J Food Prot.

[22] Khadre MA, Yousef AE, Kim J (2001). Microbiological aspects of ozone applications in food: a review. J Food Sci.

[23] Le Paulouë J, Langlais B (1999). “State-of-the-Art of Ozonation in France”. Ozone Sci. Eng. Lewis Publishers.

[24] Xu L (1999). Use of ozone to improve the safety of fresh fruits and vegetables. Food Technol.

[25] Khan NA, Paget TA (2002). Molecular tools for speciation and epidemiological studies of Acanthamoeba. Curr Microbiol.

[26] Pussard M, Pons R (1977). Morphologie de la paroikys tiqueet taxonomie du genre Acanthamoeba (Protozoa: Amoebida). Protistologica.

[27] Page FC (1988). A new key to freshwater and soil gymnamoebae.

[28] Schuster FL (2002). Cultivation of pathogenic opportunistic free-living amebas. Clin Microbiol Rev.

[29] Hewett MK, Robinson BS, Moni PT, Saint CP (2003). Identification of a New Acanthamoeba 18S rRNA Gene Sequence Type, Corresponding to the Species Acanthamoeba jacobsi Sawyer, Nerad and Visvesvara, 1992 (Lobosea: Acanthamoebidae). Acta Protozool.

[30] Schroeder JM, Booton GC, Hay J, Niszi IA, Seal DV, Markus MB, Fuerst PA, Byers TJ (2001). Use of subgenic 18S ribosomal DNA PCR and sequencing for genus and genotype identification of Acantamoeba from humans with keratitis and from sewage sludge. J Clin Microbiol.

[31] Kumar K, Tamura K, Nei K (2004). MEGA3: integrated software for molecular evolutionary genetics analysis and sequence alignment. Briefings in Bioinformatics.

[32] Kim JG, Yousef AE (2000). Inactivation kinetics of food-borne spoilage and pathogenic bacteria by ozone. J Food Sci.

[33] Hardy DJ (2010). Effect of ozone on Acanthamoeba castellanii. URMC.

[34] Cursons RT M, Brown TJ, Keys EA (1980). Effect of Disinfectants on Pathogenic Free-Living Amebas-in Axenic Conditions. App Environ Microbiol.

[35] Seidler V, Linetskiy I, Hubálková H, Staňková H, Šmucler R, Mazánek J (2008). Ozone and Its Usagein General Medicine and Dentistry. A Review Article. Prague Med Report..

[36] Hadas E, Mazur T (1993). Proteolytic enzymes of pathogenic and nonpathogenic strains of Acanthamoeba spp. Trop Med Parasitol.

[37] Jeansson S, Kvien TK (2001). Acanthamoeba polyphaga in rheumatoid arthritis: possibility for a chronic infection. Scand J Immunol.

[38] Culbertson CG (1971). The pathogenicity of soil amebas. Annu Rev Microbiol.

[39] Matin A, Jung SY (2011). Phospholipase activities in clinical and environmental isolates of Acanthamoeba. Korean J Parasitol.

[40] Kennett MJ, Hook RR Jr, Franklin CL, Riley LK (1999). Acanthamoeba castellanii: characterization of an adhesion molecule. Exp Parasitol.

[41] Rivera F, Lares F, Ramirez E, Bonilla P, Rodriguez S, Labastida A (1991). Pathogenic Acanthamoeba isolated during an atmospheric survey in Mexico City. Rev Infect Dis.

[42] Grün L, Stemplewitz B, Scheid P (2014). First report of an Acanthamoeba genotype T13 isolate as etiological agent of a keratitis in humans. Parasitol Res.

[43] Abu Kwaik Y, Gao LY, Stone BJ, Venkataraman C, Harb OS (1998). Invasion of protozoa by Legionella pneumophila and its role in bacterial ecology and pathogenesis. Appl Environ Microbiol.

[44] Chappell CL, Wright JA, Coletta M, Newsome AL (2001). Standardized method of measuring Acanthamoeba in sera from healthy human subjects. Clin Diagn Lab Immunol.

[45] Loret JF, Jousset M, Robert S, Anselme C, Saucedo G, Ribas F, Martinez A, Catalan V (2008). Elimination of free-living amoebae by drinking water treatment processes. Eur J Water Quality.

[46] Thomas V, Loret JF, Jousset M, Greub G (2008). Biodiversity of amoebae and amoebae-resisting bacteria in a drinking water treatment plant. Environ Microbiol.

[47] Khan NA (2012). Biology and pathogenesis of Acanthamoeba. Parasite &Vector.

[48] Ledee DR, Hay J, Byers TJ, Seal DV, Kirkness CM (1996). Acanthamoeba griffini. Molecular characterization of a new corneal pathogen. Invest Ophthalmol Vis Sci.

[49] Khan NA, Jarroll EL (2002). Paget, TA. Curr Microbiol.

[50] Barete S, Combes A, De Jonckeere JF, Darty A, Varnous S, Martinez V (2007). Fatal disseminated Acanthamoeba lenticulata infection in a heart transplant patient. Emerg Infect Dis.

[51] Hassan A, Farouka H, Hassanein F, Abdul-Ghanib R, Abdelhady AH (2012). Acanthamoeba contamination of hemodialysis and dental units in Alexandria, Egypt: A neglected potential source of infection. J Infect Public Health.

[52] Pankhurst CL, Johnson NW (1998). Microbial contamination of dental unit waterlines: the scientific argument. Int Dental J.

[53] Thomas V, Bouchez T, Nicolas V, Robert S, Loret JF, Levi Y (2004). Amoebae in domestic water systems: resistance to disinfection treatments and implication in Legionella persistence. J Appl Microbiol.

